# Apoptotic Cell Death Induced by Resveratrol Is Partially Mediated by the Autophagy Pathway in Human Ovarian Cancer Cells

**DOI:** 10.1371/journal.pone.0129196

**Published:** 2015-06-11

**Authors:** Fangfang Lang, Zhaoyang Qin, Fang Li, Huilin Zhang, Zhenghui Fang, Enkui Hao

**Affiliations:** 1 Department of Cardiology, Qianfoshan Hospital, Affiliated with Shandong University, Jinan, China; 2 Department of Obstetrics and Gynecology, Jinan Central Hospital, Affiliated with Shandong University, Jinan, China; 3 Department of General Surgery, Rizhao People’s Hospital, Rizhao, China; 4 Department of Health, Jinan Central Hospital, Affiliated with Shandong University, Jinan, China; 5 Central Laboratory, Jinan Central Hospital, Affiliated with Shandong University, Jinan, China; UMR INSERM U866, FRANCE

## Abstract

Resveratrol (trans-3,4,5’ –trihydroxystilbene) is an active compound in food, such as red grapes, peanuts, and berries. Resveratrol exhibits an anticancer effect on various human cancer cells. However, the mechanism of resveratrol-induced anti-cancer effect at the molecular level remains to be elucidated. In this study, the mechanism underlying the anti-cancer effect of resveratrol in human ovarian cancer cells (OVCAR-3 and Caov-3) was investigated using various molecular biology techniques, such as flow cytometry, western blotting, and RNA interference, with a major focus on the potential role of autophagy in resveratrol-induced apoptotic cell death. We demonstrated that resveratrol induced reactive oxygen species (ROS) generation, which triggers autophagy and subsequent apoptotic cell death. Resveratrol induced ATG5 expression and promoted LC3 cleavage. The apoptotic cell death induced by resveratrol was attenuated by both pharmacological and genetic inhibition of autophagy. The autophagy inhibitor chloroquine, which functions at the late stage of autophagy, significantly reduced resveratrol-induced cell death and caspase 3 activity in human ovarian cancer cells. We also demonstrated that targeting ATG5 by siRNA also suppressed resveratrol-induced apoptotic cell death. Thus, we concluded that a common pathway between autophagy and apoptosis exists in resveratrol-induced cell death in OVCAR-3 human ovarian cancer cells.

## Introduction

Ovarian cancer is one of the major leading causes of cancer-related death for females and a high rate of recurrence after surgery [1] [2]. In most cases, the diagnosis is made at late stages of the cancer, and it becomes challenging for surgical resection and recovery [2]. Thus, studies on the active ingredients of food products might provide useful alternative therapeutic approaches for this malignancy.

Resveratrol is an active ingredient from our food sources, such as grapes, peanuts, and berries, which has long been used in traditional Chinese medicine. Numerous studies have demonstrated the beneficial effects of resveratrol in cardiovascular diseases, neural diseases, obesity, and inflammatory disorders [3–5]. One of the major areas of resveratrol research is at the forefront of cancer research [6, 7]. It is well known that a high dose of resveratrol results in apoptotic cell death of ovarian cancer cells [8–10]. Several mechanisms of ovarian cancer cell death have been proposed. Phosphorylation of Cdc2-tyr15 by resveratrol treatment result in cell cycle arrest of OVCAR-3 [9]. Down-regulation of Akt/GSK and ERK signaling pathways has been shown to be critical for resveratrol-mediated cell death [10]. Recently, Lin et al. described the important role of ceramide and COX-2 in apoptotic cell death by resveratrol in OVCAR-3 [8].

Autophagy is a conservative self-degradation pathway in which cytosolic components are sequestered to lysosomes for degradation and recycling [11]. In healthy tissues, this is a process of clearing of damaged organelles. However, it is a complex process in cancer cells where it can either suppress or induce the growth of cancer cells depending on the cellular microenvironment [12].

In the present study, we investigated the potential role of autophagy in resveratrol-induced apoptotic cell death in OVCAR-3 cancer cells. We found that resveratrol treatment induced ROS generation and apoptosis, as well as activation of the autophagy pathway in OVCAR-3 cells. Inhibition of autophagy by a pharmacological inhibitor or a siRNA against ATG5 significantly attenuated resveratrol-mediated apoptotic cell death. Thus, our study established an important role of autophagy in resveratrol-induced apoptosis in human ovarian cancer cells.

## Materials and Methods

### Reagents

Resveratrol, NAC (N acetyl cysteine), chloroquine, caspase 3 assay kit, and LC3 antibody were purchased from Sigma (USA). Resveratrol was dissolved in DMSO (Sigma, USA) and was freshly prepared every time prior to cell treatment. Anti-ATG5 antibody was purchased from Beijing Biosynthesis Biotechnology, ATG5-ATG12 Complex Antibody was from AbD Serotec, and anti-cleaved caspase 3 antibody was ordered from Cell Signal technologies. siRNA against ATG5 were obtained from Shanghai GenePharma Co. Ltd. Z-VAD-FMK was purchased from R&D.

### Cell culture

OVCAR-3 and Caov-3 human ovarian cancer cell lines were obtained from ATCC (USA). The cells were cultured in RPMI 1640 (Life Technology, USA) supplemented with 10% fetal bovine serum, insulin, and penicillin–streptomycin. The cell line was grown in a CO_2_ incubator at 37°C. Human Ovarian Surface Epithelial Cells (HOSEpiC) cells were grown with Ovarian Epithelial Cell Medium provided from manufacturer (ScienCell Research Laboratories). Resveratrol treatment were described in text and figures.

### Cell viability assays

Cell viability was performed using MTT assays according to the manufacturer’s recommendations. Briefly, the cells were plated in 96-well plates and grown overnight. The cells were treated with resveratrol or other compounds as indicated for 48 h. The cells were washed with media and MTT was added 6 h before the endpoint, and the absorbance at 490 nm was measured using a microplate reader.

### Apoptosis Assay using flow cytometry

Apoptotic cell death were measured by staining with Sytox Green and Annexin V–APC (Life Technology, USA) as previously described [13].

### Cellular ROS assays

Total intracellular ROS were measured by flow cytometry using the H2DCFDA dye according to the manufacturer’s recommendation (Life Technology, USA).

### Determination of mitochondrial electron potential change

Mitochondrial membrane potential was assessed using a live cell assay with the fluorescent lipophilic cationic dye TMRE (Sigma)). This dye is a cell permeant dye that readily accumulates in active mitochondria due to their relative negative charge. After treatment OVCAR-3 cells were stained with 500 nM TMRE for 30 min at 37°C, then washed twice in medium and re-suspended in PBS. The cells were analyzed in a flowcytometer at FL2 channel and the mean intensity is plotted. itochondrial potential also analyzed by another dye JC-1. After treatment as described in the text, the fluorescent images were immediately taken using a Zeiss AX10 inverted scope (Carl Zeiss Microimaging Inc., Germany).

### Determination of 4HNE Protein adducts

The 4-HNE level in cell lysates was measured using the OxiSelect HNE Adduct ELISA Kit (Cell Biolabs) according to the manufacturer's instructions. Western blot was also performed using an anti-HNE antibody (Abcam).

### Western blotting analyses

Cells were washed with cold PBS and lysed with RIPA buffer (Sigma, USA) containing a protease inhibitor cocktail (Roche, Germany). Protein concentrations were determined using a protein assay reagent (Bio Rad, USA), and equal amounts of protein (50 μg) were loaded in each well of 4–20% or 12% SDS-PAGE. After the proteins were transferred onto a nitrocellulose membrane, the blots were blocked with 5% milk, followed by incubation with the appropriate diluted primary antibody overnight. After three washes with TBS-T buffer, the blots were incubated with appropriately diluted HRP-conjugated secondary antibodies for 2 h. The immunoblots were developed using the SuperSignal West Femto kit (Fisher Scientific, USA).

### RNA Interference

OVCAR cells were transfected with either nonspecific or ATG5 siRNA using lipofectamine (Life technologies, USA) according to manufacturer’s instruction. Cells were incubated 48 h prior to treatment with resveratrol for maximum blocking of ATG5 expression.

### Caspase 3 activity

Caspase 3 activity was measured using the caspase 3 assay kit (Sigma, USA) according to the manufacturer’s instructions. The protein concentrations were determined using the previously described method and equal amounts of protein were loaded into a 96-well plate for the caspase 3 assay.

### PARP activity

PARP activity, we used the HT Universal Colorimetric PARP assay kit from Trevigen. PARP activity is determined by the amount of PAR deposited onto immobilized histone proteins. The procedure was performed according to the recommendations of the manufacturer.

### Live cell imaging with autophagy indicator dye

Autophagic flux also was determined using Cyto-ID Autophagy Detection Kit (Enzo Life Sciences) according to manufacturer's instructions.

### Live cell imaging with fluroscent caspase substrate

Caspase activation was determined using CellEvent Caspase-3/7 Green Detection Reagent (Life technologies,USA) according to manufacturer’s instruction.

### Statistical analyses

All data were expressed as the mean with standard error of mean. Experiments were repeated four times in duplicate for all experiments. Student’s t-test or one-way ANOVA were performed as appropriate to determine the statistical significance. The significance was established at p<0.05.

## Results

### Resveratrol induced apoptotic cell death of human ovarian cancer cells in a dose-dependent manner

**Fig 1:**
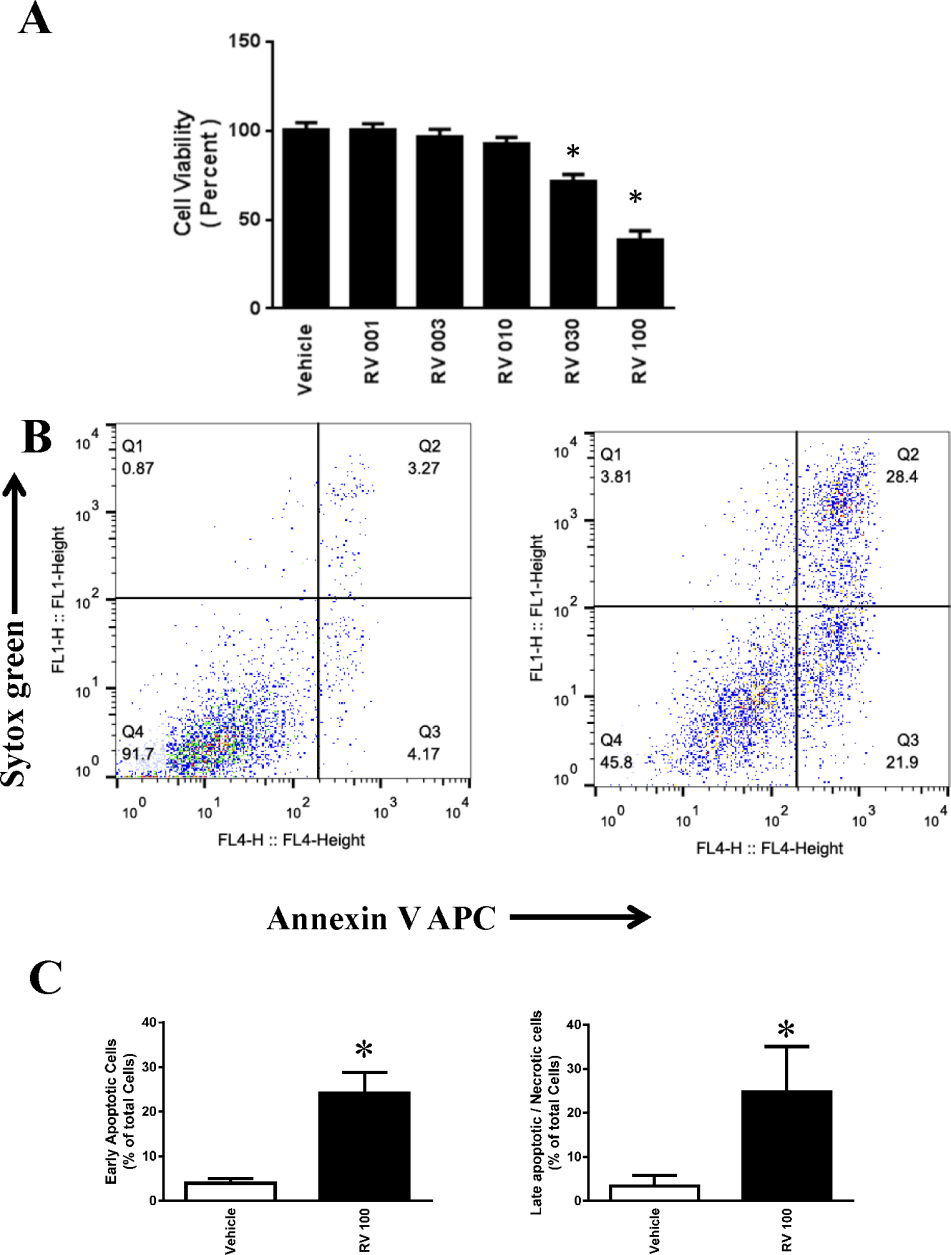
Effect of resveratrol in the cell death of ovarian cancer cells OVCAR-3. **A.** OVCAR3 cells were treated with 1 to 100 μM resveratrol for 48 h. The cell viability was determined using MTT assays. The data were obtained from an average of four independent experiments. * P<0.05 compared with vehicle control, n = 4/group. **B.** Representative flow cytometric dot plots for the measurement of apoptosis in OVCAR-3 cells. Resveratrol-induced apoptosis was measured by staining with Sytox Green dye (Y-axis, FL1) and Annexin V-APC (X-axis, FL4). **C**. Quantitative determination of early apoptotic cell death as the number of annexin V-positive cells with Sytox green-negative cells, and late apoptotic/necrotic cell death as the number of annexin V-positive and Sytox green-positive cells.

OVCAR-3 is a chemo-resistant cancer cell line established from an ovarian adenocarcinoma patient. We utilized OVCAR-3 cells as an *in vitro* cancer cell model to test the anticancer effects of resveratrol. As shown in Fig 1A, treatment with resveratrol at 1 μM to 100 μM for 48 h resulted in a dose-dependent loss of cell viability as determined using MTT assays. Treatment with 30 μM and 100 μM resveratrol showed a statistically significant loss of cell viability at 48 h.

**Fig 2:**
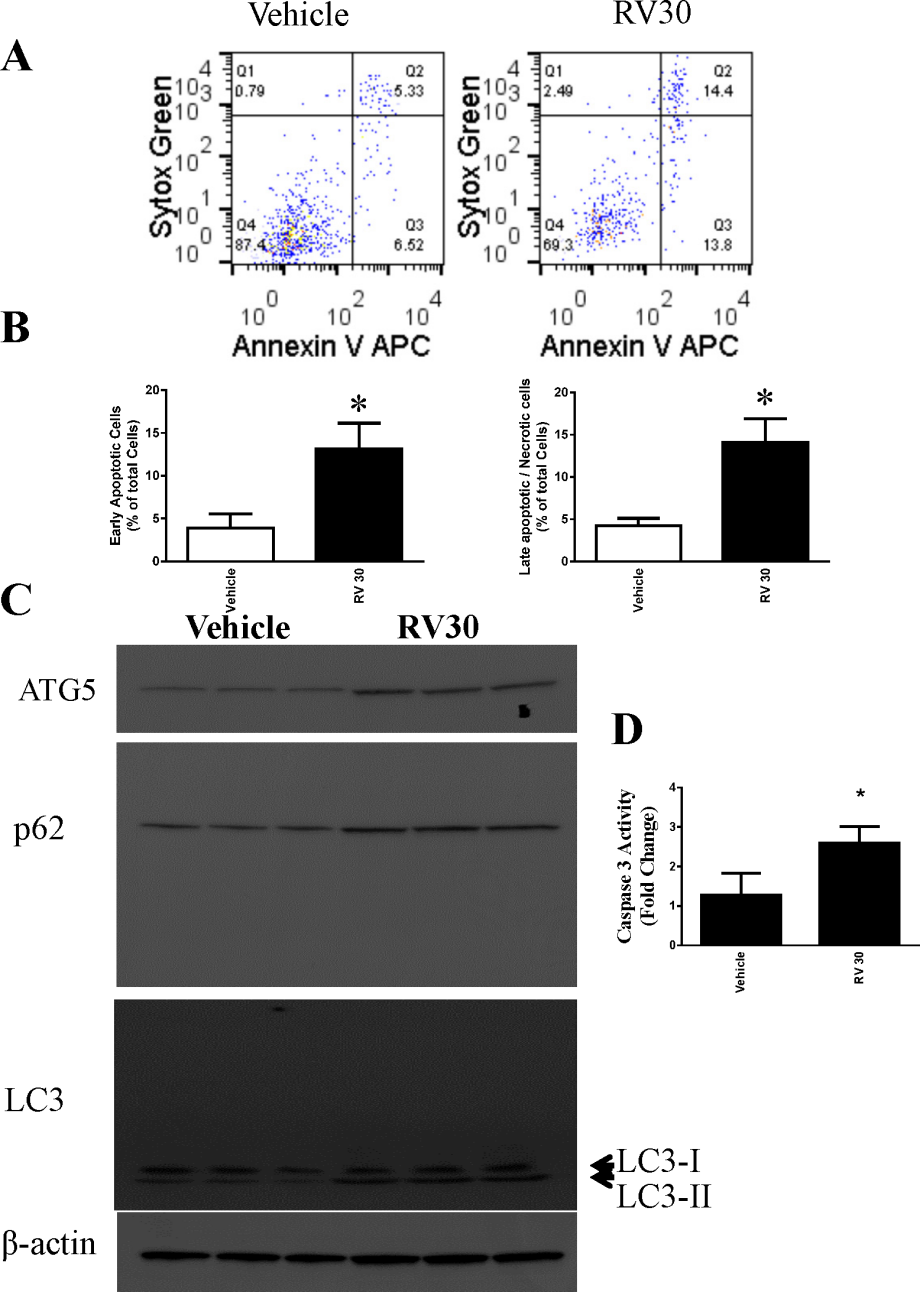
Role of autophagy in resveratrol-induced cytotoxicity in ovarian cancer cells OVCAR-3. **A.** Representative western blotting analyses of OVCAR-3 total cell lysates with ATG5, LC3, and p62 antibodies. β-actin and GAPDH were used as loading controls. Three replicate samples were loaded for each treatment group. **B.** Caspase 3 activation was measured from cell lysates and expressed as the fold change. * P<0.05 compared with the vehicle control, n = 4/group.

The effects of resveratrol on the apoptotic cell death of OVCAR-3 cells were examined by flow cytometry using Sytox Green (FL1H) and Annexin V– Allophycocyanin (FL4H). Treatment with 100 μM of resveratrol for 48 h induced significant early apoptotic and late apoptotic/necrotic cell death (Fig 1B and 1C). Treatment with 30 μM of resveratrol for 48 h also induced significant early apoptotic and late apoptotic/necrotic cell death (Fig 2A and 2B) but the effect was less compared to 100 μM.

### Resveratrol induced autophagy in human ovarian cancer cells

**Fig 3:**
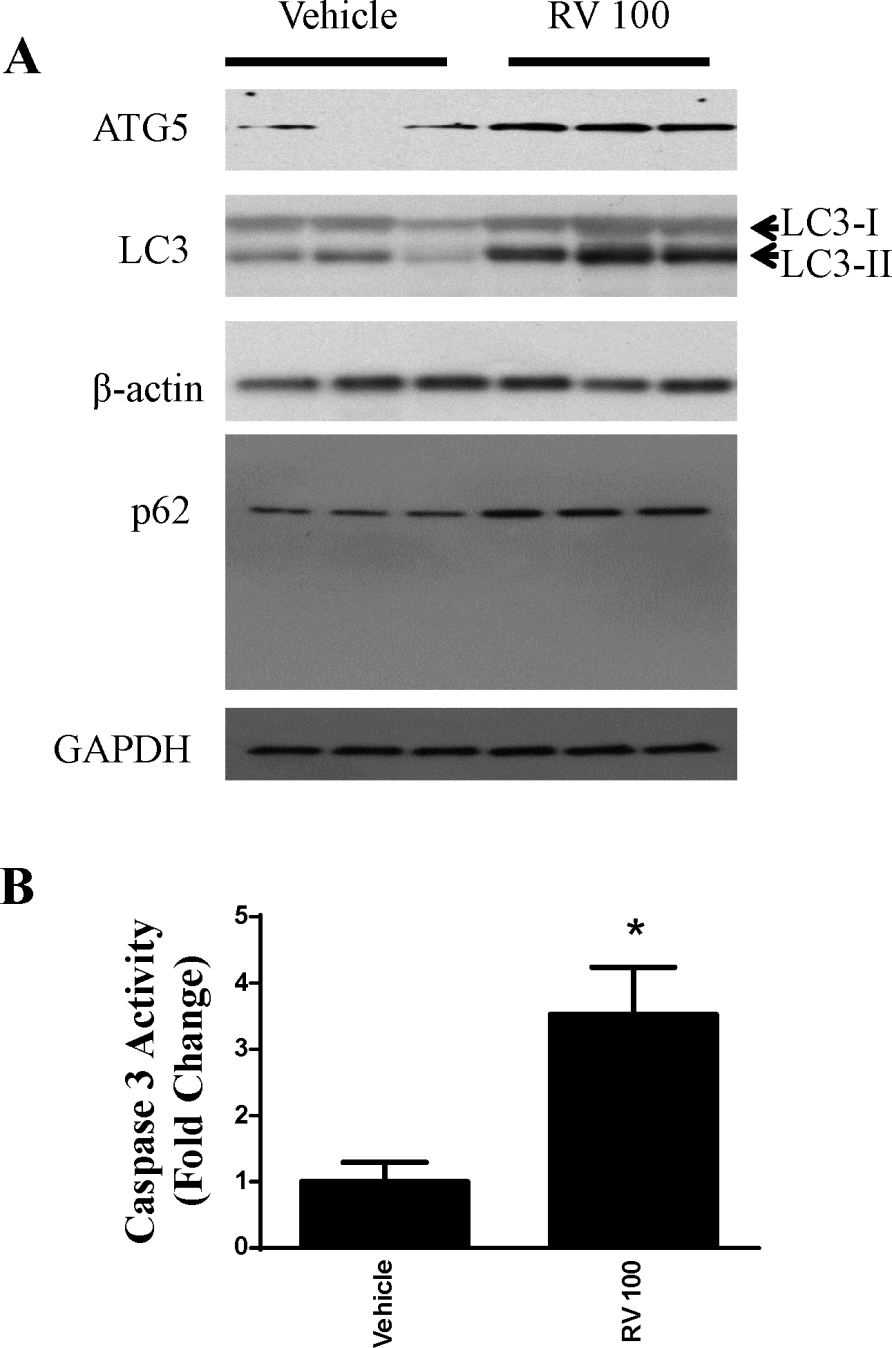
Resveratrol induced apoptosis and autophagy in OVCAR-3 cells at a lower dose. A. OVCAR-3 cells were treated with 30 μM resveratrol for 48 h, and cell death was determined by flow cytometry. B. Quantitative result of the flow cytometry. C. Representative western blotting analyses of OVCAR-3 total cell lysates with ATG5, LC3, and p62 antibodies with β-actin as a loading control.

We also evaluated the effect of resveratrol on autophagy. Key markers of autophagy were assessed. As shown in Fig 2C and Fig 3A, resveratrol ainduced LC3-II and p62 in OVCAR-3 cells. Western blotting analyses revealed an increase in the specific autophagic marker ATG5, which is essential for autophagosome formation(Figs 2A and 3A). In addition, the activity of caspase 3 was significantly increased in resveratrol-treated OVCAR-3 cells (Figs 2D and 3B).

### Resveratrol induced oxidative stress in OVCAR-3 human ovarian cancer cells

**Fig 4:**
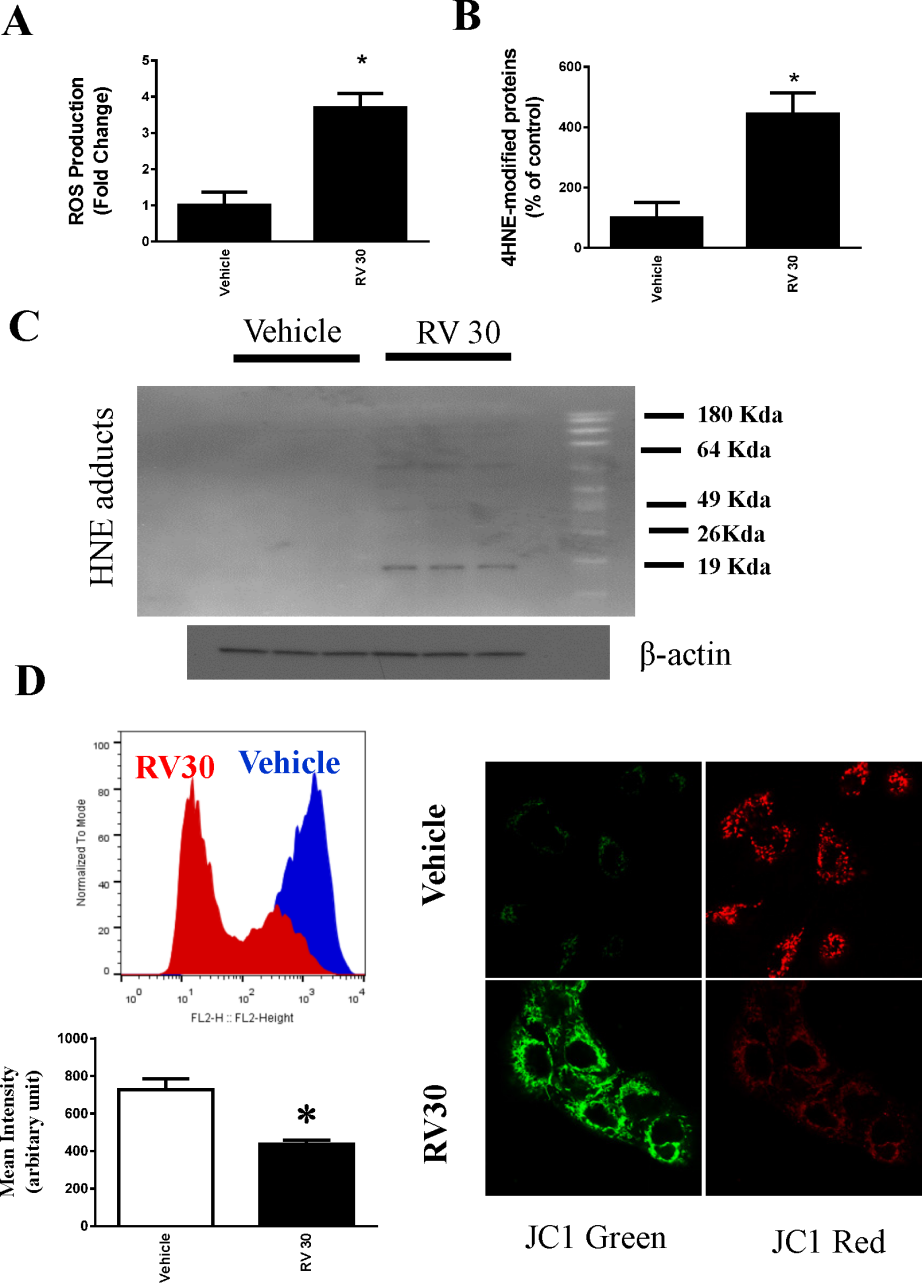
Generation of ROS by resveratrol (30 μM) in ovarian cancer cells OVCAR-3. ***A*.** Intracellular ROS of OVCAR-3 were measured by flow cytometry using H_2_DCFDA and expressed as the fold change. Resveratrol at 30 μM induced statistically significant ROS. * P<0.05 compared with vehicle control, n = 4/group. ***B*.** Oxidative stress marker 4HNE modified proteins were determined using ELISA from the cell lysates of vehicle and Resveratrol-treated (30 μM) OVCAR-3 cells. The quantified modified proteins were expressed as the % of vehicle control samples. * P<0.05 compared with vehicle control, n = 4/group. **C.** Representative Western blotting showing resveratrol induced elevation of HNE adducts. **D**. Mitochondrial electron potential change following resveratrol treatment was determined by quantitative flow cytometry and immunofluorescence.

Next, we examined whether resveratrol treatment had any effects on oxidative stress levels in the cancer cells. In the first approach, we used a cell-permeant 2',7'-dichlorodihydrofluorescein diacetate (H2DCFDA) dye to measure intracellular ROS, and found that resveratrol at 30 μM resulted in significantincrease in intracellular ROS production in OVCAR3 cells (Fig 4A). To further confirm this result, we also used a complimentary method to measure the oxidative footprint by measuring HNE protein adducts (4-Hydroxynonenal). Consistent with the intracellular ROS data, HNE protein adducts were increased significantly compared with vehicle-treated OVCAR cells (Fig 4B and 4C). Furthermore, we showed that resveratrol also significantly decreased mitochondrial electron potential (Fig 4D).

### Resveratrol similarly induced apoptosis and autophagy in human ovarian cancer cells Caov-3

**Fig 5:**
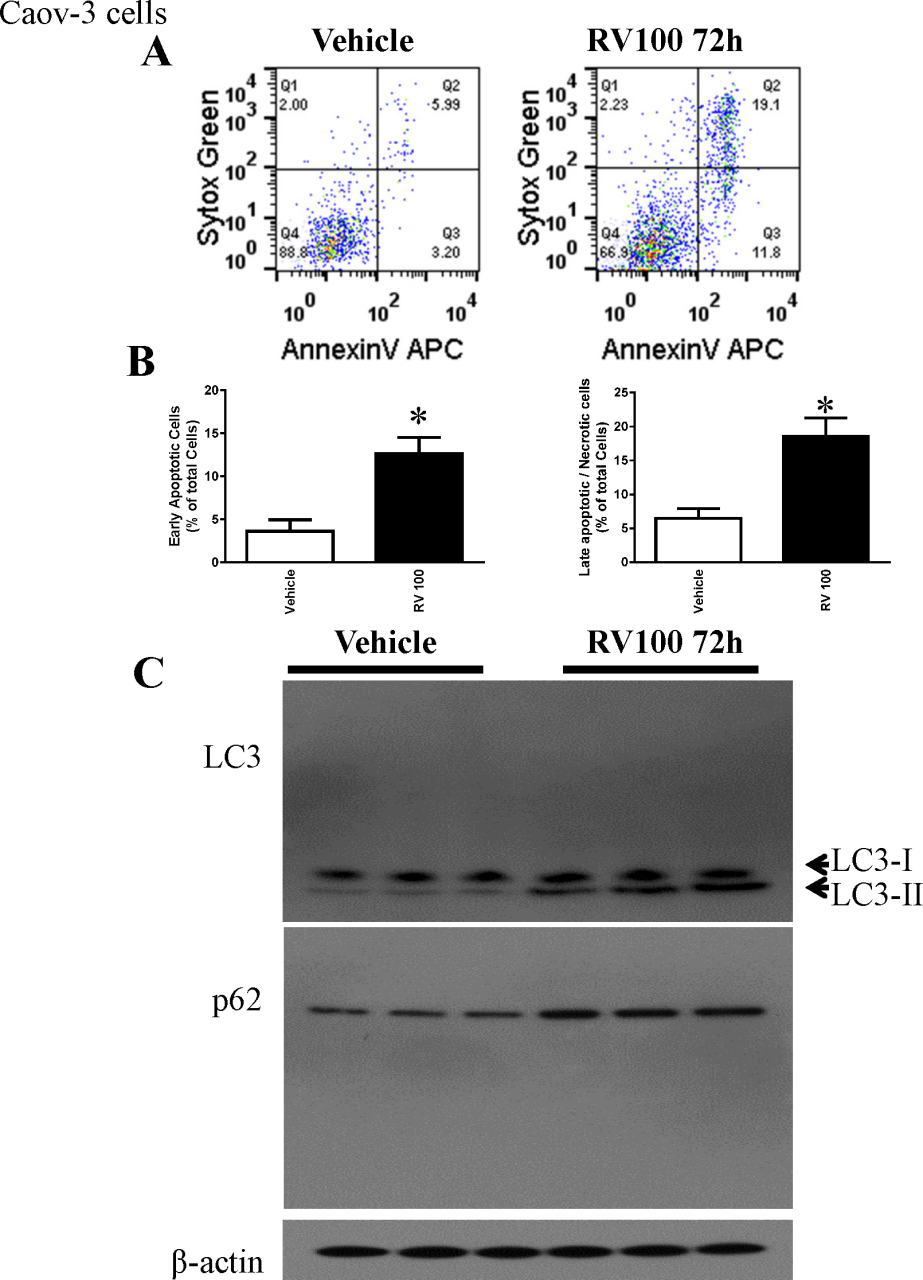
Resveratrol induced apoptosis and autophagy in Caov-3 cells. A. Caov-3 cells were treated with 30 μM resveratrol for 72 h, and cell death was determined by flow cytometry. B. Quantitative result of the flow cytometry. C. Representative western blotting analyses of Caov-3 total cell lysates with LC3, and p62 antibodies.

To determine whether the resveratrol effect is cell line specific, we utilized another human ovarian cancer cell line Caov-3. Consistent with the effect observed in OVCAR-3 cells, resveratrol also induced apoptosis and autophagy in Caov-3 cells (Fig 5). However, resveratrol at the same dose and time frame did not induce early apoptosis in primary human ovarian surface epithelial cells HOSEpiC although there is slightly increase in late apoptotic/necrotic cell death (S1 Fig).

### Pharmacological inhibition of autophagy attenuated resveratrol-induced cell death in OVCAR-3 cells

**Fig 6:**
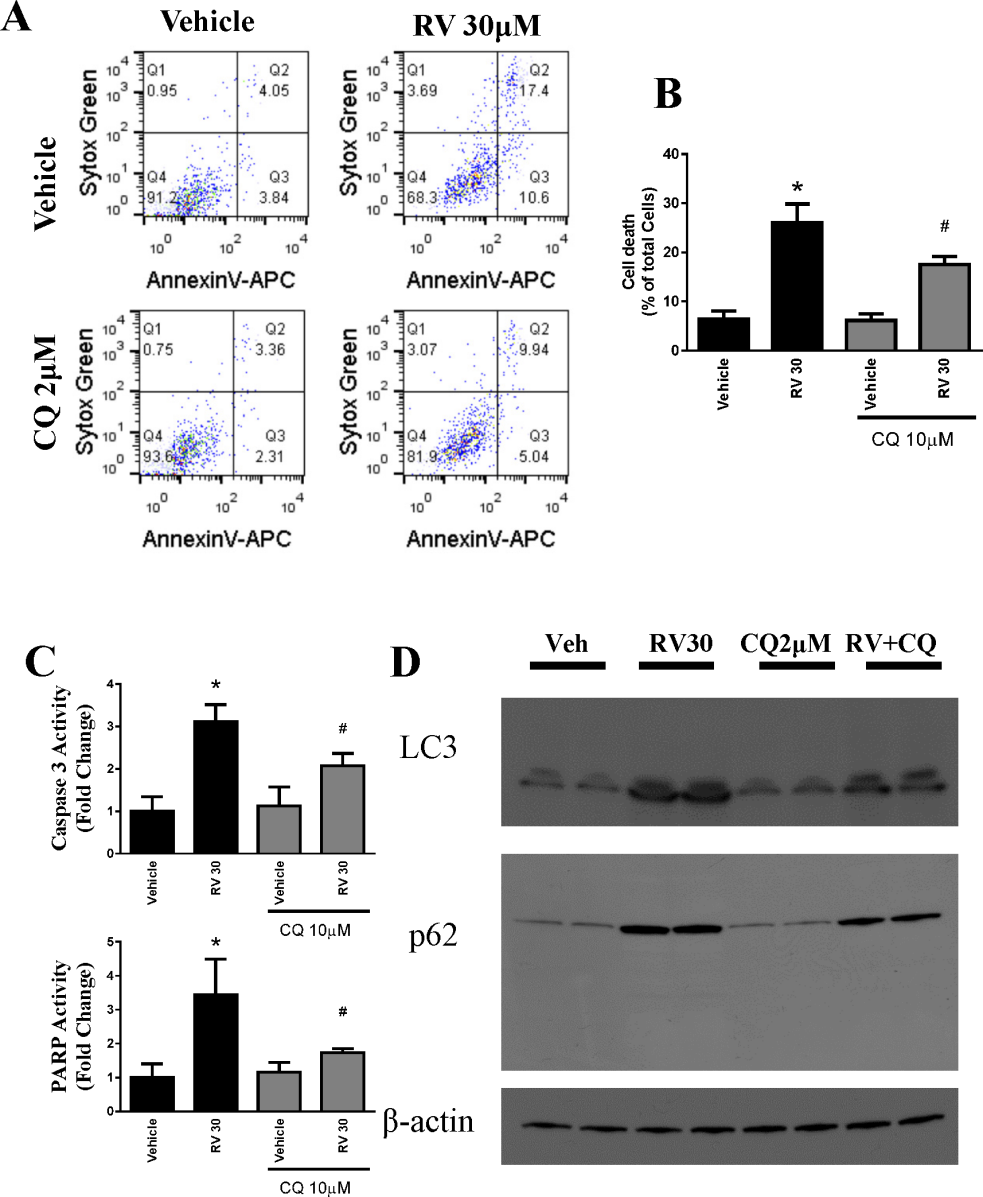
Pharmacological inhibition of autophagy protects ovarian cancer cells OVCAR-3 against resveratrol-induced cell death. *A*. Representative flow cytometry dot diagrams representing each OVCAR-3 cells. Cellular apoptosis was measured in the same method as in Fig 1. Chloroquine reduced the cell killing effect of resveratrol. B. Quantitative determination of total cell death, which was expressed as the percentage of total cells. * P<0.05 compared with vehicle control; # P<0.05 compared with resveratrol-treated samples. n = 4/group. C. Caspase 3 and PARP activities were assayed and plotted as fold change. D. Representative western blotting analyses of OVCAR-3 total cell lysates with LC3, and p62 antibodies.

To test whether autophagy is a link to apoptotic cell death, we treated OVCAR-3 cells with chloroquine (CQ), a pharmacological inhibitor of autophagy, prior to resveratrol treatment. Apoptosis and cell death were determined using Sytox Green and AnnexinV–Allophycocyanin. As shown in Fig 6A, cell death by resveratrol at 30 μM was significantly attenuated by pretreatment with 2 μM of CQ. Resveratrol significantly reduced the total cellular death by autophagy inhibition. also analyzed resveratrol induced apoptosis markers caspase 3 activity and PARP activity and both were attenuated by CQ treatment (Fig 6C). In addition, we observed that resveratrol-induced autophagosome markers LC3II and p62 were also attenuated by CQ treatment (Fig 6D).

### Inhibition of autophagy by ATG siRNA ameliorated resveratrol-induced cell death in OVCAR-3 cells

**Fig 7:**
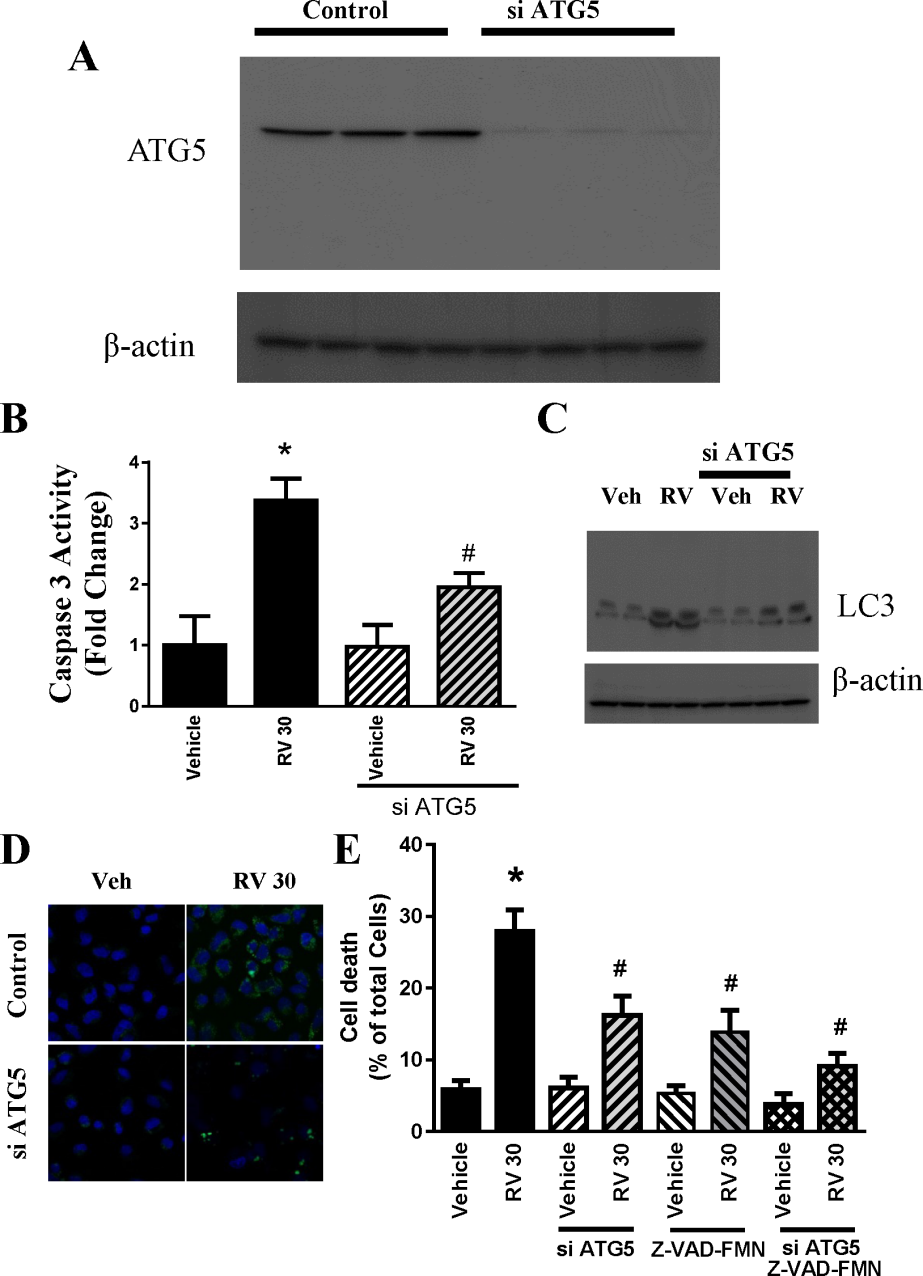
RNA interference of ATG5 attenuated resveratrol-induced cell death and caspase activity. A. ATG5 protein expression was determined from cellular lysates obtained from OVCAR-3 cells loaded with either control random siRNA or ATG5 siRNA. B. Caspase 3 activities were determined from cellular lysates obtained from OVCAR-3 cells loaded with either control random siRNA or ATG5 siRNA. The activities were expressed as the fold change. * P<0.05 compared with vehicle control, # P<0.05 compared with resveratrol-treated samples. n = 4/group. C. Representative western blotting analyses of OVCAR-3 treated with vehicle (Veh) or Resveratrol at 30 μM (RV) with and without siATG5 and total cell lysates were anlyzedwith LC3 and β-actin D. Live cell image from confocal microscope(with 20X objective) using Cyto-Id dye loaded for 15 minutes after treatment with resveratrol at 30 μM in OVCAR-3 cells transformed with random or ATG5 siRNA. E. Quantitative determination of cell death in OVCAR cells using flow cytometry. Resveratrol-induced cell death is attenuated in siATG5-treated and siATG5 plus Z-VAD-FMK (50μM) treated OVCAR-3 cells. * P<0.05 compared with vehicle control; # P<0.05 compared with resveratrol-treated samples. n = 4/group.

ATG5 is the hallmark of autophagy and a key player in the formation of autophagosomes. In addition to the pharmacological inhibitor, we used a genetic approach by knocking down ATG5 expression using a specific siRNA and examined its effect on resveratrol-induced cancer cell death. As shown in Fig 7, cell death by resveratrol (30μM) was reduced from 27.9% to 16.22% by ATG5 siRNA. A similar pattern was also observed in caspase 3 activity and LC3 western blot (Fig 7B and 7C). The efficacy of siRNA technology was confirmed using western blotting analyses, which indicated a significant reduction of ATG5 protein levels (Fig 7A). Interestingly, we found that the cells treated with ATG5 siRNA in combination with Z-VAD-FMK(50μM) are more significantly protected from resveratrol-induced cell death compared to those treated with either inhibitor alone (Fig 7E). We also employed new live cell imaging technology using Cyto-ID dye for autophagy flux and the above results were confirmed.

**Fig 8:**
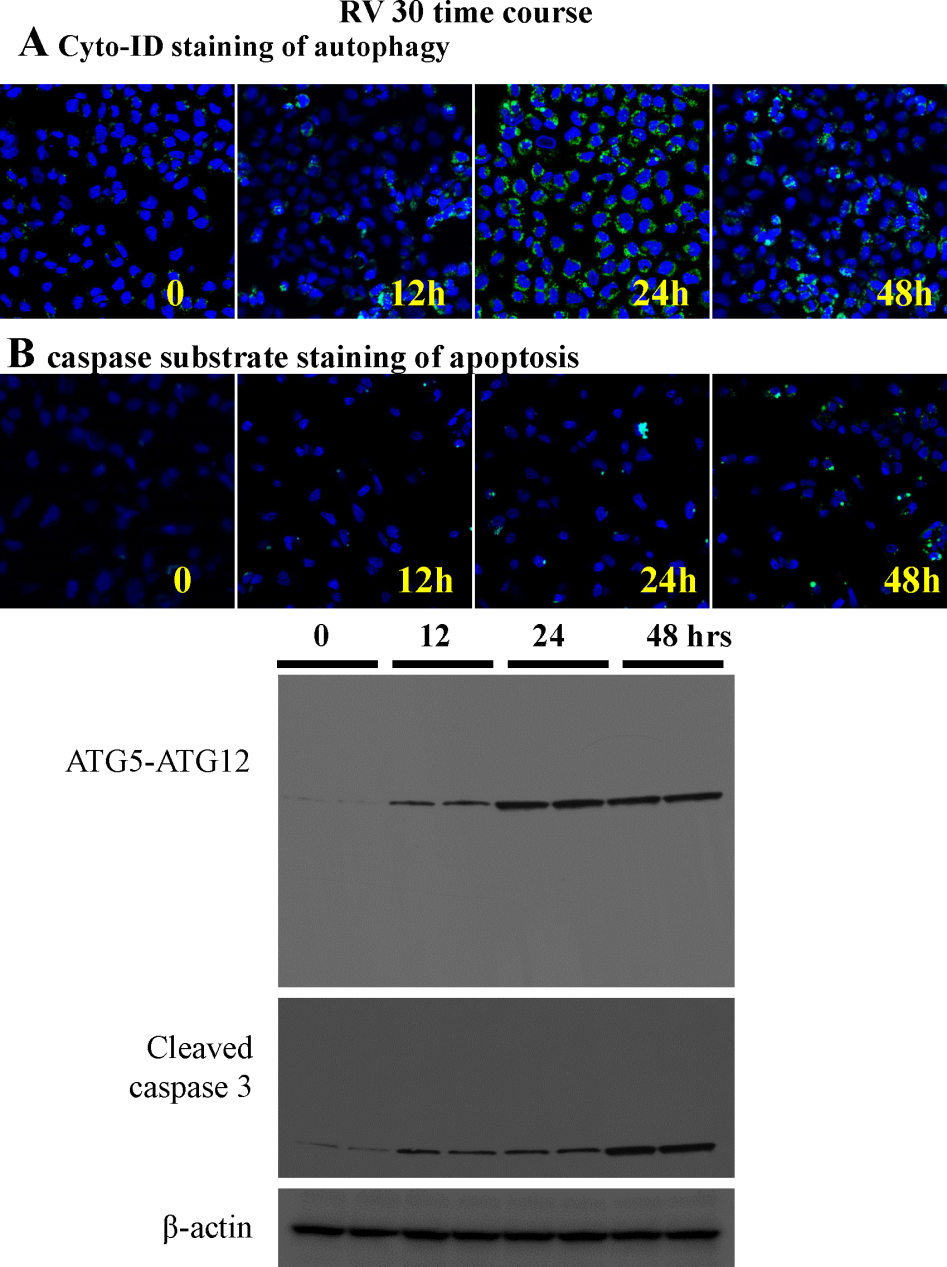
The time course of autophagy and apoptosis induction by 30 μM resveratrol treatment. A. Live cell imaging by confocal microscopy (with 10X objective) with Cyto-ID dye in OVCAR-3 cells treated with 30 μM resveratrol at 0, 12, 24 and 48 hour time points. B. Live cell imaging by confocal microscopy (with 10X objective) with caspase 3 green detection dye in OVCAR-3 cells at 0, 12, 24 and 48 hour time points. C. OVCAR-3 cells were treated with 30 μM of resveratrol for 12 h, 24 h, and 48 h. Western blotting of total cell lysates were performed with anti-ATG5-ATG12, LC3, and caspase 3 antibodies. No cleavage of ATG5 was observed. The results shown represent an experiment, in which each time-point of treatment was duplicated.

We used live cell imaging for autophagic flux and caspase 3 substrate dye (marker for early apoptosis) using microscopy (Fig 8A and 8B). It was evident that autophagic flux was initiated earlier (12h) than apoptosis (24h) when treated with 30M resveratrol, which is in agreement of the aforementioned ATG5 siRNA data. We also performed a time course study with Western blot analyses using two sets of samples from independent experiments at each time points (Fig 8C) and found that autophagy was activated earlier than apoptosis, suggesting that autophagy is upstream of apoptosis in the ovarian cancer cells treated with resveratrol. However there are overlap between the two processes which is a typical pattern of any cancer cell population.

## Discussion

In this study, we demonstrated that resveratrol-induced cell death in human ovarian cancer cells was mediated by both apoptosis and autophagy. Resveratrol induced significant intracellular ROS generation and oxidative stress in cancer cells, which resulted in initiation of the cell death pathway. The hallmarks of autophagy, LC3 and ATG5, were both upregulated in resveratrol-treated cancer cells. We have also shown that pharmacological inhibition of autophagy by chloroquine attenuated resveratrol-induced cell death in OVCAR-3, and similar results were obtained by blocking ATG5 expression using siRNA.

Several recent studies have shown some intriguing pathways that mediate resveratrol-induced apoptotic cell death. Vergara et al. described the role ERK signaling and AKT/GSK pathways in apoptotic cell death using a proteomic approach [10]. In another elegant study, Lin et al. demonstrated that resveratrol-induced apoptosis shared similar pathways with ceramide-induced COX-2-dependent apoptosis in ovarian cancer cells [8]. ere, we have shown that the apoptotic cell death of OVCAR-3 induced by resveratrol is far more complex and autophagy plays a critical role in this process.

One of our key observations was the production of intracellular ROS and oxidative stress in resveratrol-induced cell death in OVCAR-3 cells. ROS is involved in modulating various pathways, including the ERK signaling pathway [14–16]. Intriguingly, ROS could induce autophagy in resveratrol-treated OVCAR-3 cells [17]. Resveratrol is known to have beneficial antioxidant effects on healthy cells [18], but these antioxidant activities have different consequences in highly proliferating cancer cells. Antioxidants, including resveratrol, have been shown to contribute to ROS-induced cell death in other cancer cell types [19, 20]. Our data suggest that ROS production by resveratrol in OVCAR-3 cells is associated with cancer cell apoptosis, which is consistent to the findings obtained in previous studies focused on colorectal and colon carcinoma cells.

In general, autophagy inhibits the induction of apoptosis, and apoptosis-associated caspase activation turns off the autophagy process. However, there are many cases where both of these processes occur simultaneously or co-activates to ROS production induced both autophagy and apoptosis in cancer cells [21]. Autophagy itself can induce cell death, a process known as autophagic cell death [22]. It has also been reported that induction of autophagy facilitates the activation of apoptosis [23]. Puissant et al found that both autophagy and apoptosis are involved in resveratrol-induced cell death in a myelogenous leukemia cell line [24–26]. In MCF7 breast cancer cells, resveratrol induced beclin-1 dependent and independent autophagy [27].Here, we also observed both caspase dependent and independent pathway exists for resveratrol induced cell death in ovarian cancer cells. Either caspase or autophagy inhibitors were unable to restore resveratrol induced cell death. One of the key proteins involved in this process is the tumor suppressor protein p53 and its protein levels in the cytosol modulate activation or inhibition of autophagy[28, 29]. Nuclear translocation of p53 is also important for apoptotic induction. It has been shown that p53 is crucial for resveratrol-mediated cell death in OVCAR-3 cells [8]. Other common mediators of both autophagy and apoptosis are several BH3-only (BCL-3 homology 3) proteins. During apoptosis, BH3-only proteins directly interact with the BCL-2 family, thereby silencing the anti-apoptotic role and stimulating apoptosis. Several BH3-only proteins, such as BAD and BNIP3 can also promote autophagy by competitive inhibition between Beclin1 and apoptotic proteins BCL-2 [30–32]. Interestingly, both BCL2 and Bad have been shown to be dysregulated in OVCAR-3 cells [8].

The involvement p62 in resveratrol induced cell death in leukemia cell line is critical[25]. We also observed induction of p62 in ovarian cancer cells in response to resveratrol treatment.

In our study, we found that inhibition of autophagy attenuates (but not completely abolishes) resveratrol-induced ovarian cancer cell death. We also performed a time course study, and found that resveratrol activates autophagy earlier than apoptosis, suggesting that autophagy is upstream of apoptosis. Together, these data support that resveratrol induces apoptosis partially through autophagy. We agree that some more classic mechanisms are likely also involved in resveratrol induced apoptosis.

Autophagy is a crucial survival mechanism for cancer cells under strict conditions to meet their nutrient requirements. Concurrent inhibition of autophagy during anti-cancer therapy is beneficial in urological cancer [33]. In this study, we used the late stage autophagy inhibitor chloroquine and found that autophagy mediates OVCAR-3 cell death induced by resveratrol, which was confirmed by transfection of a siRNA against ATG5. Our data suggest that it would not be beneficial to treat ovarian cancer with an autophagy inhibitor in addition to resveratrol. Combination of resveratrol with an autophagy trigger would possibly enhance the anticancer effect. This would be an interesting and important topic worth further study. Importantly, previous studies in esophageal squamous cell carcinoma demonstrated the opposite effect by inhibition of autophagy in resveratrol-induced cell death [34]. This might be partially due to the different dosages and types of cancer cells used in this study, and autophagy is a complex process that functions like a double-edged sword when harnessed as a way to fight against cancer [35–39].

## Supporting Information

S1 Fig Resveratrol did not induce apoptosis in healthy ovarian surface epithelial cells. Flow cytometric analyses of Human Ovarian Surface Epithelial Cells (HOSEpiC) cells, which were grown with Ovarian Epithelial Cell Medium provided from manufacturer (ScienCell Research Laboratories). Resveratrol treatment was performed at 100μM for 48 hours. Cells were then stained with AnnexinV and sytox green for early apoptosis and late apoptosis/necrotic cell death markers respectively. Quantitative determination of flow cytometry data demonstrated no significant difference in early apoptic cell death markers but little significant increase in late apoptosis/necrotic cell death markers. * P<0.05 compared with vehicle control; n = 3/group.
(TIFF)
