# Supplementary figures and images for: Apoptotic Cell Death Induced by Resveratrol Is Partially Mediated by the Autophagy Pathway in Human Ovarian Cancer Cells
Source: PLoS One. 2015 Jun 11;10(6):e0129196. doi: 10.1371/journal.pone.0129196 (PMC4466135; doi:10.1371/journal.pone.0129196)

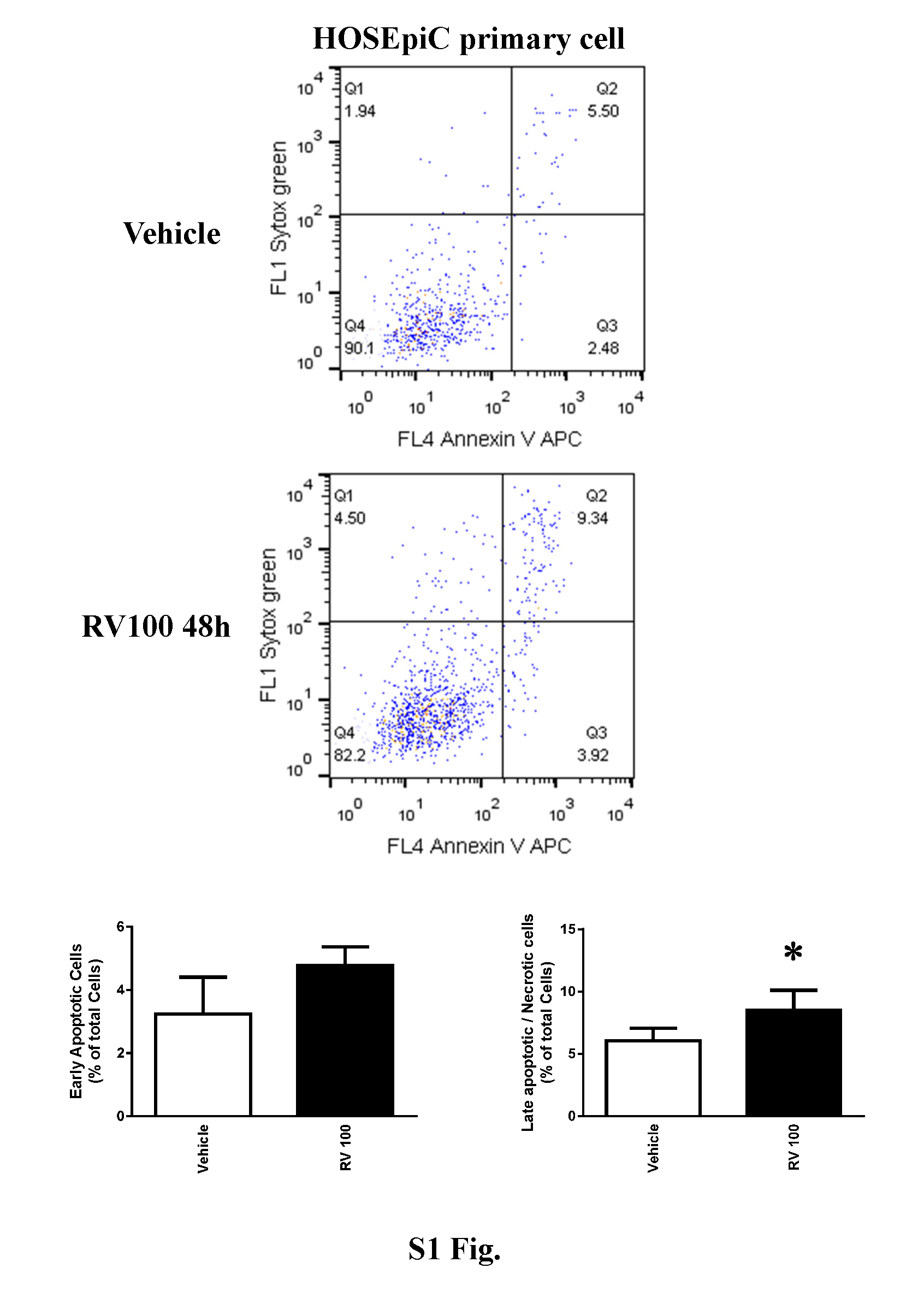

Supplement: S1 Fig — Flow cytometric analyses of Human Ovarian Surface Epithelial Cells (HOSEpiC) cells, which were grown with Ovarian Epithelial Cell Medium provided from manufacturer (ScienCell Research Laboratories). Resveratrol treatment was performed at 100μM for 48 hours. Cells were then stained with AnnexinV and sytox green for early apoptosis and late apoptosis/necrotic cell death markers respectively. Quantitative determination of flow cytometry data demonstrated no significant difference in early apoptic cell death markers but little significant increase in late apoptosis/necrotic cell death markers. * P<0.05 compared with vehicle control; n = 3/group. (TIFF) [file pone.0129196.s001.tiff]
